# Risk Factors for Non-Adherence and Loss to Follow-Up in a Three-Year Clinical Trial in Botswana

**DOI:** 10.1371/journal.pone.0018435

**Published:** 2011-04-25

**Authors:** Deborah A. Gust, Barudi Mosimaneotsile, Unami Mathebula, Balladiah Chingapane, Zaneta Gaul, Sherri L. Pals, Taraz Samandari

**Affiliations:** 1 Division of HIV/AIDS Prevention, Centers for Disease Control and Prevention (CDC), Atlanta, Georgia, United States of America; 2 Botswana-USA Partnership, Centers for Disease Control and Prevention (CDC), Gaborone and Francistown, Botswana; 3 Division of Tuberculosis Elimination, Centers for Disease Control and Prevention (CDC), Atlanta, Georgia, United States of America; University of Delhi, India

## Abstract

**Background:**

Participant non-adherence and loss to follow-up can compromise the validity of clinical trial results. An assessment of these issues was made in a 3-year tuberculosis prevention trial among HIV-infected adults in Botswana.

**Methods and Findings:**

Between 11/2004–07/2006, 1995 participants were enrolled at eight public health clinics. They returned monthly to receive bottles of medication and were expected to take daily tablets of isoniazid or placebo for three years. Non-adherence was defined as refusing tablet ingestion but agreeing to quarterly physical examinations. Loss to follow-up was defined as not having returned for appointments in ≥60 days. Between 10/2008–04/2009, survey interviews were conducted with 83 participants identified as lost to follow-up and 127 identified as non-adherent. As a comparison, 252 randomly selected adherent participants were also surveyed. Multivariate logistic regression analysis was used to identify associations with selected risk factors. Men had higher odds of being non-adherent (adjusted odds ratio (AOR), 2.24; 95% confidence interval [95%CI] 1.24–4.04) and lost to follow-up (AOR 3.08; 95%CI 1.50–6.33). Non-adherent participants had higher odds of reporting difficulties taking the regimen or not knowing if they had difficulties (AOR 3.40; 95%CI 1.75–6.60) and lower odds associated with each year of age (AOR 0.95; 95%CI 0.91–0.98), but other variables such as employment, distance from clinic, alcohol use, and understanding study requirements were not significantly different than controls. Among participants who were non-adherent or lost to follow-up, 40/210 (19.0%) reported that they stopped the medication because of work commitments and 33/210 (15.7%) said they thought they had completed the study.

**Conclusions:**

Men had higher odds of non-adherence and loss to follow-up than women. Potential interventions that might improve adherence in trial participants may include:targeting health education for men, reducing barriers, clarifying study expectations, educating employers about HIV/AIDS to help reduce stigma in the workplace, and encouraging employers to support employee health.

**Trial Registration:**

ClinicalTrials.gov NCT00164281

## Introduction

Non-adherence and loss to follow-up in a clinical trial threatens the validity of conclusions about the intervention. The Botswana Isoniazid Preventive Therapy (IPT) clinical trial was conducted between November 2004 and July 2009. It was a double-blinded, randomized, placebo-controlled clinical trial to determine whether isoniazid taken daily for 36 months was more effective in protecting against tuberculosis (TB) in HIV-infected adults compared to the standard-of-care in which isoniazid was taken daily for six months. The researchers reported that participants receiving 36 months of IPT had half the risk of TB compared to participants receiving the 6-month regimen [1]. During the conduct of the trial, a sub-study was conducted to assess the causes of non-adherence.

While IPT reduces the incidence of TB disease among HIV and TB co-infected individuals [2], adherence may be difficult because of the long duration of prophylaxis. Typically, patients with chronic conditions have poorer medication adherence than those with acute conditions [3]. It is important to understand factors associated with non-adherence so that high adherence can be maintained or low adherence improved [3]–[6] since adherence to effective treatment improves health outcomes [7], [8].

Non-adherence to medication regimens is observed in clinical trials and is common under routine program conditions. Participants may wish to stop taking the medication or cease returning for clinic appointments. In order to better understand what factors affected non-adherence in our study, we conducted a sub-study in which we identified two distinct groups of non-adherent participants:participants who refused to take the study medication but agreed to return for study visits and participants who were lost to follow-up. Demographic characteristics and other risk factors of these two groups were compared against those who remained adherent in the Botswana IPT clinical trial. As an earlier analysis of trial data found that being on antiretroviral therapy (ART) was associated with better IPT adherence [9], we included ART use as a risk factor.

## Methods

### Study population

Potential participants for the clinical trial were recruited from 5 public health clinics in Gaborone and 3 in Francistown [1], [9]. Participants in the trial were ≥18 years of age and infected with HIV and all were required to pass a 20-question study comprehension quiz before enrollment. Subsequent to enrolment in the trial, ART was provided to eligible study participants through routine government services for management of their HIV infection. Participants were required to take isoniazid or placebo and one tablet of vitamin B6 daily for three years. Those who also initiated ART typically took 2 pills twice daily in addition to the study medications. Study nurses provided bottles of study medication and interviewed participants monthly, provided reminder cards for their visits and performed pill counts with the participants on a quarterly basis. The expected visit window for the monthly pharmacy refill was 7 days early or 14 days late in the 30-day study month. The visit window was included to allow some flexibility to the study participants and the time frame chosen was based upon practical considerations specific to the study area. If a study participant did not attend a visit, nurses and retention officers attempted to reach her/him, her/his designated friend or family member by mobile phone at first and subsequently paid visits to her/his home. Participants stopping study medication because of adverse events or refusal to continue taking their pills were invited to remain in the study and were followed off study medication for the remainder of the trial.

**Figure 1:**
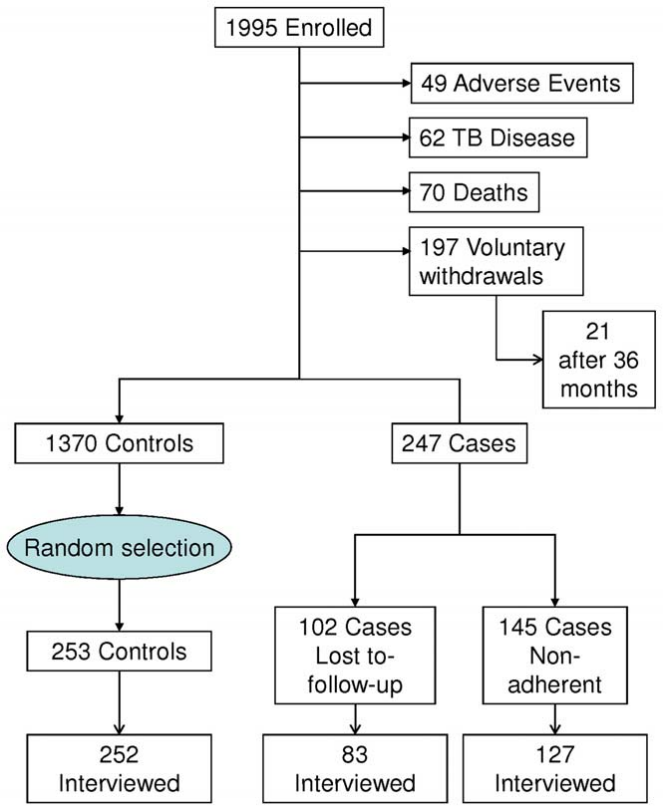
Derivation of cases and controls for the adherence sub-study from the cohort of HIV-infected persons enrolled in the Botswana Isoniazid Preventive Therapy Trial, 2004–2008. Case-Non-adherent was defined as not taking the study medication due to unwillingness to take any more study medication but continuing to attend quarterly visits and seen at the last expected visit. Case-LosT to follow-up was defined as a participant who was still expected to take the study medication and receive monthly medication refills but missed the last visit by≥60 days. A control was defined as a participant who continued to be on study medication and was last seen within the expected visit window which was 7 days early or 14 days late in the 30-day study month. The median number of days since the last visit of those cases who were lost to follow-up was 396 days (range 91–1196), and 48 days (range 6–116) for the cases who were non-adherent. As the sub-study was conducted between 10/2008 AND 4/2009, 21 PARTICIPANTS HAS ALREADY COMPLETED THE RQUIRED 36 MONTHS OF OBSERVATION AND HAD VOLUNTARILY WITHDRAWN.

Participants in the current sub-study were divided into cases and controls (Fig. 1). Information about frequency of returns for scheduled medication pick-up visits (pharmacy refill) was used to identify controls and two types of cases. The types of cases were those who continued attending clinic visits but were non-adherent (case non-adherent) and those lost to follow-up (case lost to follow-up). Non-adherence was defined as 1) refusal to take any more study medication, 2) agreeing to attend quarterly visits and 3) seen at the last expected visit. Loss to follow-up was defined as 1) a participant who was still expected to take the study medication and receive monthly medication refills and 2) missed the last visit by ≥60 days. A control was defined as 1) a participant who continued to be on study medication and 2) last seen within the expected visit window. Participants who completed the first 6 months of daily isoniazid and subsequently missed study medications or scheduled visits were invited to resume taking their medication and were considered adherent for the sub-study if they agreed to and returned sooner than 60 days. Between 10/2008–04/2009, 102 participants were identified as lost to follow-up, 145 were identified as non-adherent. As a comparison an equal number of controls were randomly selected from 1370 adherent participants. Random selection is superior to other methods of sampling [10], [11]. Participants who developed severe adverse events, TB disease or died were excluded from consideration as either cases or controls. According to the study protocol, research staff were not permitted to contact participants who had voluntarily withdrawn.

### Procedures

Rates of pharmacy refill visits among enrolled participants were determined using a criterion of attending ≥80% of visits within six 6-month periods (Table 1). As part of the main IPT trial, without prior notification, urine samples were collected from 200 randomly selected participants in the 36-months and 50 participants in the placebo arm who continued to return to the clinic by study nurses and analyzed by non-study staff for isoniazid metabolites [12] on three occasions approximately one year apart. Results of isoniazid metabolite testing were reported in aggregate by the study statistician so that double-blinding was maintained.

**Table 1:** Pharmacy refill rates and urine testing for isoniazid metabolites among participants of the Botswana Isoniazid Preventive Therapy Trial, 2004–2008.

Period after enrolment (months)	Participants enrolled at beginning of period	Attending ≥80% of clinic visits			% with detectable urine isoniazid metabolites in 36H arm*
		6H	36H	Total	
1–6	1995	86%	85%	85%	-
7–12	1945	87%	80%	84%	-
13–18	1870	79%	77%	78%	80%^†^
19–24	1808	79%	76%	77%	79%^‡^
25–30	1755	79%	75%	77%	74%^§^
31–36	1712	79%	77%	78%	-
**1–36**	**1995**	**78%**	**77%**	**78%**	**N/A**

*Unannounced, urine samples were collected in 2006, 2007 and 2008 from 200 randomly selected participants receiving isoniazid who returned for refills. The numbers of participants and their median (range) month in the study were:194 at month 13^†^ (5–22), 202 at month 22^‡^ (12–30), 195 at month 30^§^ (21–36). Abbreviations: 6H = six months of isoniazid daily followed by placebo; 36H = 36 months of isoniazid daily.

In order to better understand factors associated with non-adherence and loss to follow-up, a third party (a private company in Botswana) was hired to contact sub-study participants and carry out the focus group discussions, interviews and surveys. This third party was enlisted in order to reduce the risk that study participants would be less than forthcoming in their responses to questions and also to facilitate finding lost to follow-up participants. Focus group discussions and individual interviews were first conducted to provide supplemental information and to help finalize a survey that was administered during the second phase of the study (the complete questionnaire is available upon request). Native Setswana-speaking persons moderated the focus group discussions and interviews.

#### Phase 1: Group discussions and individual interviews

Group and individual interviews were conducted prior to finalizing the survey instrument to assure that relevant questions were included. Purposeful sampling, or selecting participants based on their ability to provide information on the relevant subject [13], was used to choose 42 participants using the criterion that they could be categorized as a case non-adherent or a case lost to follow-up as defined above. Groups were divided by sex to ensure that the females would have a chance to express themselves freely due to the sensitive nature of the topic. There were 3 groups of women and 2 groups of men. A semi-structured focus group moderator and individual interview guide was prepared to elicit information about participants' thoughts and opinions about the trial as well as reasons for non-adherence. The guide queried reasons for joining the trial, knowledge of TB, why isoniazid prophylaxis is important for persons infected with HIV, barriers to trial participation, adequacy of information provided about the trial, perception of the care and treatment provided by trial staff, benefits of being in the trial, and reasons participants stopped taking the study pills.

#### Phase 2: Survey

The survey instrument was interviewer-administered and consisted primarily of close-ended questions. Participants (247 cases and 253 controls, see Fig. 1) were contacted by phone to set up a time to have the survey administered at an office in Gaborone or Francistown and were given the option to have the survey administered over the phone. Focus group and individual interview participants were not excluded from participating in the survey. Reimbursements were deposited to the participants' bank accounts or given directly to those who came to the offices. Survey items covered the same topics as the moderator's guide.

### Ethics Statement

Participants were provided explanations about the sub-study, were told that the information they provided would be confidential, and were informed that their participation was voluntary so they could refuse to answer any questions or leave the focus group at any time. Verbal, not written, consent was obtained for this sub-study because the risks were minimal and participants had already provided written consent for the parent clinical trial. The ethics committees agreed that verbal consent was sufficient. For the survey and interviews, if the potential participant refused to participate and consent, this was recorded. All participants designated as cases who participated in the group discussion or individual interviews received a monetary reimbursement (approximately U.S. $8.00) for their time and transportation costs. Ethical approval was obtained from the Botswana Ministry of Health's Human Research Development Committee and the Centers for Disease Control and Prevention's Institutional Review Board (see Protocol S1 and Checklist S1). The clinical trial is registered at clinicaltrials.gov, NCT00164281.

### Analysis

#### Qualitative Analysis

*Nvivo*, a qualitative data analysis program, was used to organize the data and code themes from transcribed discussions and interviews. A “grounded theory” method was used for data analysis, an inductive approach where theory is generated from the data. Two researchers independently read and coded two of the 12 transcripts in order to assess inter-coder reliability. The researchers read and discussed their coding to reach consensus. Cohen's kappa coefficient for 22 questions in these transcripts prior to their resolutions ranged from 0.82 to 0.93. One researcher (DG) performed the final coding of all discussions and interviews. Quotes were selected on the basis of their clear representation of the key themes.

*Quantitative Analysis.* We conducted bivariate and multiple regression analyses to test for differences in several variables of interest between controls and cases. For bivariate analyses, chi-square tests were used and, in the case of the continuous variable, age, a *t*-test was used. For multiple regression analyses, two logistic regression models were constructed; one model assessed differences between controls and non-adherent cases and the second model assessed differences between controls and cases lost to follow-up. All of the independent variables with a *P*-value <0.25 in bivariate analysis were entered into the multiple logistic regression models. Independent variables included in both models were age, sex, whether the participant was employed, income, education, time to get to clinic, whether the participant drank alcohol, the main reason for joining the trial, whether the participant received ART, CD4^+^ T-lymphocyte cell count at enrolment, whether the participant understood study expectations, whether the participant had difficulties with the regimen, whether the participant believed isoniazid was dangerous to her/his health, and whether the participant believed that more information would have helped her/him remain adherent. ART use and baseline CD4^+^ cell count data were taken from the main clinical trial database and used as independent variables. In both models adjusted odds ratios (AORs), 95% confidence intervals (95%CI) and a test for the overall significance of the model variables with more than 2 levels (Type 3 analysis of effects test) were computed to assess pairwise comparisons. Data for visit attendance for pharmacy refills were also taken from the main clinical trial database and used for descriptive purposes.

## Results

Between November 2004 and July 2006, 1995 participants were enrolled in the clinical trial. Overall, the clinical trial had a very good adherence rate during the 3 years of follow-up with 78% of participants adherent to at least 80% of their scheduled visits, more than three-quarters of randomly selected participants having detectable isoniazid metabolites in their urine (Table 1) and 91% having reached a known study endpoint by 36 months of follow-up.

### Sub-study

From the dataset of October 8, 2008, we identified 247 cases and from a pool of 1370 controls, we randomly selected 253 controls (Fig. 1). At the time of this selection there had been 4,740 person-years of observation which is approximately two-and-a-half years of follow-up per participant. The median number of days since the last visit of those cases who were lost to follow-up was 396 days (range 91–1196), and 48 days (range 6–116) for the cases who were non-adherent.

### Phase 1-Group Discussions/Interviews

Cases discussed their views on several topics related to adherence to the study regimen. There were five in-person group discussions consisting of 18 individuals (11 females, 7 males) and seven individual interviews (3 females, 4 males; one face-to-face and six via telephone) for a total of 25 individuals. Regarding their treatment by clinic staff, cases strongly expressed that the clinical trial staff provided them with information about their health, treated them with respect, and imbued them with a sense of empowerment. Themes associated with barriers to trial participation included, for example, competing commitments, side effects, and relocation (Table 2). Cases also provided suggestions as to how to improve retention in the trial, for example having more dispersed clinics and communicating requirements related to the trial to the broader community. Other suggestions are presented in Table 3.

**Table 2:** Examples of group discussion and interview quotations from trial participants who were non-adherent to study medication or lost to follow-up in the Botswana Isoniazid Preventive Therapy Trial, 2004–2008.

Domain of Inquiry	Theme	Examples
Reasons joined trial	Benefit to self	“It seemed better to prevent than to contract TB.”
	Benefit to others	“…by becoming a participant I could be able to advise other young people and discuss issues like the importance of programs like IPT with them.”
	Referred	“…I tested positive. And they referred me to the IPT office where I started taking the treatment.”
Knowledge of TB	Symptoms	“It is a cough that is easily spread to other people and you lose a lot of weight.”
	Transmission	“…if you spit on the ground, it can spread to others easily.”
Facilitators to trial participation	Health-related	“I managed to prevent TB and I'm happy because I do not have TB. I know my status. They also check my CD4 count every time I go for monthly check ups.”
Barriers to trial participation	Competing commitments	“The reasons were work commitments. My job was a barrier to taking the pill but the medication treated me well.”
	Side effects	I always felt like vomiting and my eyes were always itching because of the pills.”
	Started ART	“I was taking a lot of tablets and I was always thinking I will die…so I decided to stop these ones (isoniazid).”
	Stigma	“They (Batswana) still discriminate against people on the trial and that discrimination is what makes people drop out of the trial..”
	Relocate	“My job contract came to an end and I had to relocate to my home village”
	Lack of staff	“…the barriers… the one I can think of is the lack of staff.”
	Transport	“When you are far from the clinic, the transport to the clinic becomes a problem.”
	Inconsistent	“…when I was taking the trial medication then I started with ART I asked whether I should continue with the isoniazid and they said I could just stop isoniazid as its really not a problem.”
Treatment by clinic staff	Respect	“Every time I did not understand, I asked and they made sure they explained clearly in order for me to understand better.”
	Empower	“They made me realize that I can move forward. I was able to take ART without being reluctant and this trial made me build a home for my family because of my confidence.”
	Information	“We learned a lot about TB. We have learned how one can be infected, how it can be treated, how dangerous it is and many more other things.”

**Table 3:** Suggestions to improve retention from participants who were non-adherent or lost to follow-up in the Botswana Isoniazid Preventive Therapy Trial, 2004–2008.

Suggestions
More dispersed clinics
*“We need to have a number of dispersed clinics so that people who are residing in rural areas get the medical services they need at the right times.”*
*“They should increase study sites because some live far away from towns so that they become nearer to us and we don*'*t become lazy to visit.”*
Communication of trial requirements to broader community
*“Even the bosses should be told about this program so that tomorrow when people ask for permission for these visits every month, they should know what is going on.”*
More staff
*“The staff should be increased so that the patients may be assisted quickly.”*
Convenient times for appointments
*“They (future participants) should be able to agree on a time that would also be suitable for them.”* *“My request is that some health care workers in this trial should work on weekends.”*
Communication
*“You should communicate with us by taking our phone numbers to check how we are doing.”*
Shorter trial
*“Three years is also too long, some people may get tired or hopeless and stop taking the medication”*

### Phase 2-Survey

Among the 500 cases and controls selected for this survey study, 462 completed the survey for a response rate of 92.4% (462/500). The response rate for the controls (252/253 or 99.6%) was higher than for the non-adherent (127/145 or 87.6%) and lost to follow-up cases (83/102 or 81.4%) (Fig. 1). Due to work or personal schedules, 76% (351/462) of cases and controls chose to have the survey administered over the phone.

### Bivariate analysis-associations with non-adherence and loss to follow-up

Compared to controls, the case non-adherent group was younger (t = 58.2, *P*<0.0001), and had a greater proportion of men (χ^2^(1) = 5.7, *P* = 0.017), persons with higher education (χ^2^(2) = 3.6, *P* = 0.170), persons who drank alcohol (χ^2^(1) = 4.4, *P* = 0.036), persons who initiated ART (χ^2^(1) = 1.70, *P* = 0.192), persons who did not understand what was expected when they joined the trial (χ^2^(1) = 3.3, *P* = 0.071) and persons who experienced any difficulty with the regimen (χ^2^(1) = 21.9, *P*<0.0001). Compared to controls, the case lost to follow-up group was younger (t = 49.7, *P* = 0.0001) and had a greater proportion of men (χ^2^(1) = 14.4, *P*<0.0001), persons with a higher income (χ^2^(2) = 4.2, *P* = 0.120), persons with a higher education (χ^2^(2) = 11.5, *P = *0.003), persons who reported a longer time to get to the clinic (χ^2^(2) = 8.9, *P* = 0.012), persons who drank alcohol (χ^2^(1) = 6.81, *P* = 0.009), persons who had not initiated ART (χ^2^(1) = 2.81, *P* = 0.094), persons who did not understand what was expected when they joined the trial (χ^2^(1) = 9.38, *P* = 0.002), and persons who experienced any difficulty with the regimen (χ^2^(1) = 5.4, *P* = 0.020).

### Multivariable regression analysis-associations with non-adherence and loss to follow-up

Compared to controls, non-adherent participants had greater odds of being male (AOR 2.24; 95%CI 1.24–4.04) and having difficulties with the regimen or not knowing if they had difficulties (AOR 3.40; 95%CI 1.75–6.60), and had lower odds of being older (AOR 0.94 for each year of age; 95%CI 0.91–0.98) (Table 4). Participants lost to follow-up had higher odds of being male (AOR 3.08; 95%CI 1.50–6.33) and having a secondary education (AOR 2.55; 95%CI 1.10–5.91). However, secondary education was not statistically significant in the Type 3 analysis of the multivariate model.

**Table 4:** Risk factor analysis using multivariate logistic regression analysis for non-adherence and loss to follow-up in a cohort of HIV-infected adults enrolled in the Botswana Isoniazid Preventive Therapy Trial, 2004–2008.

Demographic Characteristic or Risk Factor		Adherent	Non-adherent		Lost to follow-up	
			N (%)§	AOR (95% CI)	N (%)§	AOR (95% CI)
Age (years)	Mean±SD	37±10	34±7	0.94 (0.91, 0.98)*	35±6	0.99 (0.95, 1.04)
Sex						
	Female	199 (79)	86 (68)	Referent	48 (58)	Referent
	Male	53 (21)	41 (32)	2.24 (1.24, 4.04)*	35 (42)	3.08 (1.50, 6.33)*
Employed						
	Yes	161 (64)	80 (63)	–	54 (66)	–
	No	90 (36)	47 (37)	–	28 (34)	–
Income per month (Pula)						
	0–900	96 (52)	41 (43)	–	30 (41)	Referent
	901–2000	58 (31)	35 (37)	–	23 (32)	1.26 (0.63, 2.52)
	>2000	31 (17)	19 (20)	–	20 (27)	1.03 (0.42, 2.53)
Education						
	Primary or less	99 (41)	38 (31)	Referent	18 (22)	Referent
	Secondary	127 (52)	74 (60)	0.97 (0.51, 1.85)	50 (62)	2.55 (1.10, 5.91)
	Tertiary	18 (7)	12 (10)	1.05 (0.39, 2.78)	13 (16)	2.72 (0.68, 10.80)
Time to get to clinic (minutes)						
	<30	73 (29)	33 (26)	–	36 (44)	Referent
	30–60	144 (58)	76 (60)	–	32 (39)	0.64 (0.33, 1.24)
	>60	32 (13)	18 (14)	–	14 (17)	1.61 (0.61, 4.26)
Do you drink/take alcohol?						
	No	227 (90)	104 (83)	Referent	64 (79)	Referent
	Yes	25 (10)	22 (17)	1.55 (0.76, 3.17)	17 (21)	1.26 (0.51, 3.09)
Main reason for joining the trial						
	Prevent TB	167 (66)	78 (61)	–	53 (64)	–
	Other†	85 (34)	49 (39)	–	30 (36)	–
Started on antiretroviral therapy (ART)						
	Yes	133 (53)	76 (60)	Referent	35 (42)	Referent
	No	119 (47)	51 (40)	0.67 (0.41, 1.10)	48 (58)	1.18 (0.63, 2.21)
Baseline CD4^+^ T cell count						
	≥200 cells/mm^3^	173 (69)	83 (66)	–	56 (68)	–
	<200 cells/mm^3^	76 (31)	43 (34)	–	26 (32)	–
When I enrolled in the trial, I understood what was expected of me						
	Agree	205 (82)	94 (74)	Referent	54 (66)	Referent
	Disagree/Don't know	45 (18)	33 (26)	1.65 (0.93, 2.90)	28 (34)	1.87 (0.93, 3.75)
I didn't have any difficulties with the regimen						
	Agree	231 (93)	92 (75)	Referent	69 (84)	Referent
	Disagree/Don't know	18 (7)	30 (25)	3.40 (1.75, 6.60)*	13 (16)	1.46 (0.58, 3.66)
The isoniazid medication may be dangerous to my health						
	Agree	48 (21)	26 (23)	–	14 (20)	–
	Disagree/Don't know	180 (79)	85 (77)	–	57 (80)	–
More information about TB would help me stay with the medication						
	Agree	219 (89)	114 (91)	–	74 (91)	–
	Disagree/Don't know	26 (11)	11 (9)	–	7 (9)	–

Notes. Missing values are not included; AOR = adjusted odds ratio, SD = standard deviation.

*Overall significant, i.e. *P*<0.05 by Type 3 analysis of effects.

‘–’ = Not included in multiple regression model because *P*-value>0.25 in bivariate analysis.

† Examples of other responses included:“to receive TB education”, “receive free medical care”, “to prevent TB”, “to receive incentives for taking part”, “recruited or advised to do so”, “because I am HIV positive”, “to help my country”, “to help the study succeed.”

§ For the variable Age, this column reflects the mean ± standard deviation of the mean (SD).

### Self-reported reasons for stopping the medication

Among cases, 40/210 (19.0%) reported that they stopped the medication because of work commitments (non-adherent 18.9%, lost to follow-up 19.3%) and 33/210 (15.7%) said they thought they had completed the study (non-adherent 17.3%, lost to follow-up 13.3%) (Table 5). Side effects were noted by a larger proportion of non-adherent cases than those lost to follow-up (15.8% versus 6.0%) and relocation was noted by a larger proportion of cases lost to follow-up than those who were non-adherent (18.1% versus 7.9%).

**Table 5:** Self-reported reasons non-adherent and loss to follow-up cases stopped taking isoniazid medication in the Botswana Isoniazid Preventive Therapy Trial.

Reasons	Non-adherent N (%)	Lost to follow-up N (%)
Work commitment	24 (18.9)	16 (19.3)
Personal doctor told me to stop because of medical problems including side effects of the study medication	10 (7.9)	6 (7.2)
Side effects of the study medication (but personal doctor did not tell me to stop)	20 (15.8)	5 (6.0)
Stigma associated with being in the trial	3 (2.4)	2 (2.4)
Relocated too far away to keep appointments	10 (7.9)	15 (18.1)
Not enough transport money (does not include relocating)	2 (1.6)	6 (7.2)
Completed the study (though had not)	22 (17.3)	11 (13.3)
Pregnant	3 (2.4)	3 (3.6)
Take too many pills	1 (0.8)	2 (2.4)
Lost/forgot	7 (5.5)	2 (2.4)
Other†	23 (18.1)	4 (4.8)
No reason provided	2 (1.6)	11 (13.3)
Total	127 (100%)	83 (100%)

† Examples of other responses included:social problems, religious beliefs, boyfriend threw the pills away, sister flushed the pills down the toilet, pills were stolen, miscommunication, long lines at the clinic, pills increased appetite.

## Discussion

Adherence and retention in clinical trials are important issues because participant non-adherence and loss to follow-up can compromise study results. Generally speaking, patients are less adherent to treatment when they feel well, such as when taking prophylactic treatment, than they are for a symptomatic condition [14], [15]. We report on non-adherence and loss to follow-up in 1,995 HIV-infected adults enrolled in a 3-year prophylaxis trial in two Botswana cities. Based upon the pharmacy refill criterion for adherence (i.e.,≥80% attendance to study medication refill visits), despite a prolonged 36-month period of prophylaxis, participants in our trial had a 78% adherence rate which is similar to the 69–86% rates reported by other clinical trials that provided 6 months of IPT [16]–[18]. Using the urine isoniazid metabolite criterion as an assessment of adherence, participants in the 36-month Botswana study had a 74–80% adherence rate which is comparable to the 45–80% rates reported in two other IPT clinical trials of shorter duration [16], [18]. We conducted a case-control sub-study in which we interviewed 83 participants who had been lost to follow-up and 127 participants who were non-adherent to study medication but still attending visits and compared their demographics and responses to specific questions with those of 252 adherent participants. Factors associated with non-adherence or loss to follow-up included age, male sex and difficulties with the regimen. Additional information from participants identified side effects and competing commitments such as work, relocation, and the belief that they had completed the study as contributions to non-adherence or loss to follow-up.

In the case-control sub-study, men had twice and thrice the odds of women to be non-adherent or lost to follow-up, respectively. While we were unable to identify published studies examining adherence to prophylactic therapy in large numbers of HIV-infected adults in sub-Saharan Africa, adherence to ART has been assessed in such populations. Among enrollees of ART programs in Côte d'Ivoire and Kenya, men were more likely than women to be lost to follow-up [19], [20]. In South Africa, based upon pharmacy records, women were more likely than men (54% vs 49%, *P *<0.001) to take ≥80% of their ART [21], although this phenomenon is not limited to South Africa [22]. While reasons for the increased risk of non-adherence in African men attending public health clinics are poorly understood, employment circumstances and frequency and amount of alcohol use are two possible explanations. The employment rate in men in the Botswana trial was higher than women (82% vs. 63%), but the type of employment and the work hours of men may have made adherence more difficult. Indeed interview and focus group participants noted that taking time off from work for study visits was often difficult. Widespread heavy alcohol use has been reported in Botswana communities, particularly in men (31% vs. 17% in women) [23] and was paralleled by observations from the IPT trial (data not shown). Reasons why HIV-infected men are more likely to be non-adherent need to be identified in future studies and should guide approaches taken to improve their adherence.

Non-adherent participants had more than three times the odds of reporting difficulties with the regimen or not knowing if they had difficulties than controls (rates reported were 25% in non-adherent cases vs. 7% in controls) and although not significant in the multivariable model, participants lost to follow-up also had higher rates (16%) of reporting these difficulties or not knowing. Some discussion group and interview participants reported that the study medication made them sick, lose weight, feel dizzy or tired or made their body ache. It is well established that side-effects can greatly influence an individual's willingness to adhere to therapy [24], [25]. Based upon trial records, none of these individuals suffered adverse effects of the study medication. However it is possible that they may have had mild but irritating symptoms which they did not report to the study staff. This may represent reporting bias in that patients are more likely to self-report adverse effects of a medication than clinicians are to record them [26] or it may represent an excuse for stopping the medication. Younger age was also associated with non-adherent participants as has been commonly reported in the medical literature and also for HIV-infected persons [27]–[29].

Some associations that were significant in bivariate analysis were not significant in the adjusted analyses but bear mentioning:distance from clinic, alcohol use, level of education, and understanding the requirements of the study. All four factors have been reported as barriers to adherence [30], [22], [31], [32]. It may be that these factors were not independently associated with non-adherent or lost to follow-up participants in our study because of small numbers, differences in question comprehension, or confounding with other variables. For instance, alcohol use may not have been significant in the adjusted analysis because the question did not ask about the frequency and quantity of alcohol consumed. Notably, a relatively large proportion of both groups (17% non-adherents and 13% lost to follow-up) reported stopping the medication because they believed they had completed the study. This may indicate that either nursing staff did not adequately explain study requirements to the participants or that the participant did not fully comprehend what was being explained. Alternatively, as posited by nursing staff, some cases may have used this option as an excuse because all participants were required to pass a comprehension test before enrollment.

Our study had several potential limitations. First, data were cross-sectional, so we cannot state that the significant factors led to a participant becoming non-adherent or lost to follow-up. Second, responses to survey questions were self-reported which may have resulted in some bias in answering questions about non-adherence, although we believe this was minimized since the study was conducted by an independent contractor and not members of the clinic staff. Third, the clinical trial staff did not have control over the independent administration of ART to study participants during their changing disease course over the 3-year study. As HIV-infected persons widely regard ART as life-sustaining and as ART consists of many additional pills and also have significant adverse effects, they may have complicated adherence to study medication. Fourth, we did not assess the personalities of the participants which could have potentially been a factor in their adherence to study medication. Fifth, because we were unable to contact 197 participants who had voluntarily withdrawn at the time the sub-study was begun, the lack of information about them may have biased our sub-study. Finally, all of the relevant and important variables may not have been captured in our survey (e.g., peer or family social support). The strengths of this study are the inclusion of group discussions and interviews to supplement the survey data, clear definitions of non-adherence and loss to follow-up, and the very high survey response rate.

Studies have shown that participant satisfaction with the clinic staff can contribute to adherence [32]. In our study, discussion group and interview participants overwhelmingly praised their treatment from the clinic staff in a number of areas. Moreover, the majority of cases responding to the survey agreed that they were treated well by the clinic staff to the degree that the variables could not be included in the multiple regression model because the number of negative responses was too small. Suggestions from the discussion group and interview participants as to what would improve their experience ranged from adding weekend clinic hours to adding more clinics in different locations, but none related to the way they were treated by clinic staff. For future clinical trials, potential interventions that might improve adherence in trial participants, even for non-modifiable factors like sex, include targeting health education for men, reducing barriers (e.g. offering more clinics in different locations if possible and weekend hours, making visits brief and convenient), clarifying study expectations, educating employers about HIV/AIDS to help reduce stigma in the workplace, and encouraging employers to support employee health.

## Supporting Information

Protocol S1 A Randomized, Placebo-Controlled Study of Limited vs. Continuous Isoniazid Tuberculosis Preventive Therapy for HIV-infected Persons in Botswana.
(PDF)

Checklist S1 CONSORT checklist for Protocol S1.
(DOC)
