# Supplementary material for: Risk Factors for Non-Adherence and Loss to Follow-Up in a Three-Year Clinical Trial in Botswana
Source: PLoS One. 2011 Apr 25;6(4):e18435. doi: 10.1371/journal.pone.0018435 (PMC3081815; doi:10.1371/journal.pone.0018435)
Supplement: Protocol S1 — A Randomized, Placebo-Controlled Study of Limited vs. Continuous Isoniazid Tuberculosis Preventive Therapy for HIV-infected Persons in Botswana. (PDF) [file pone.0018435.s001.pdf]

A Randomized, Placebo-Controlled Study of

Limited vs. Continuous Isoniazid Tuberculosis Preventive Therapy

for HIV-infected Persons in Botswana

Protocol Team

Taraz Samandari MD, PhD, Samba Nyirenda MD, Barudi Mosimaneotsile RN, MPH,

Tefera Agizew MD M Phil, Zegabriel Tedla MD, Thabisa Sibanda MB ChB,

Themba L. Moeti MBBS, M Sc, Julius Mboya MD MSc, Howard J. Moffat MB ChB, FRCP,

Deborah A. Gust PhD MPH, Lorna Bozeman MS, Peter H. Kilmarx MD, Charles D. Wells MD

The BOTUSA Project

Epidemiology Unit, Ministry of Health, Botswana

Division of TB Elimination, Centers for Disease Control and Prevention, USA

Division of HIV/AIDS Prevention, Centers for Disease Control and Prevention, USA

Gaborone City Council

Francistown City Council

Technical and Supportive Services, Ministry of Health, Botswana

Protocol Overview

The Government of Botswana is taking important steps to reduce the impact of the severe HIV epidemic on its population. There are plans to expand voluntary counseling and testing (VCT) services nationwide to enable persons living with HIV infection (PLWH) to learn their serostatus, plan for the future, and to access services for appropriate care. In April 1999, the Government also implemented a pilot program to prevent the maternal to child transmission of HIV (MTCT) through 1) the use of zidovudine during pregnancy and labor; 2) the provision of infant formula to limit breast feeding; and 3) the administration of zidovudine to the infant. The Government has also prioritized the development and provision of a "package of care" for PLWH to prevent opportunistic infections, such as tuberculosis (TB) and other bacterial infections. Toward this end, in March 2000, the Government approved a pilot program to prevent the progression of *M. tuberculosis* infection to active TB through the use of isoniazid (isoniazid preventive therapy, or IPT).

Objective: To determine if continuous IPT is superior to limited among PLWH.

Specific objectives include:

1. Determine the proportion of PLWH eligible for IPT who have a positive TST, abnormal chest radiograph, and active TB;
2. Estimate subject compliance with limited vs. continuous INH;
3. Determine if continuous IPT is superior to limited IPT to prevent active TB and death in PLWH;
4. Ascertain the causes of morbidity and mortality among subjects;
5. Quantify the generation of INH resistance in subjects who develop active TB during or after IPT;
6. Identify risk factors for the development of TB in persons taking limited vs. continuous INH, including demographic characteristics, compliance, TB

exposure, TST results, chest radiograph findings, CD4, and total lymphocyte counts).

7. Genetic analysis will be performed on mycobacterial isolates in order to identify clusters which suggest recent transmission, as well as to estimate the relative burden of recent transmission compared with reactivation of latent TB infection.

Design: Randomized, blinded, two-arm comparative trial in Gaborone and Francistown, Botswana. Subjects will be accrued over one year and followed for 36 months.

Primary Endpoints: Active pulmonary or extrapulmonary TB (definite, probable, & possible TB)

Secondary Endpoints:

1. The combined endpoints of: definite and probable TB; active TB and deaths in which TB was not excluded as the cause; active TB and deaths
2. Adverse events
3. Death

Outcome Variables: CD_4 T-lymphocyte cell count, PPD positivity

Sample Size: 2,000 subjects (total – 1,000 each arm)

Eligible Participants:

1. HIV-infected
2. Age ≥18 years
3. Tuberculin skin test positive or negative
4. Laboratory:

   Hgb ≥6.5 gm/dl

   Neutrophil count ≥1,000 cells/mm^3

   Platelets ≥75,000/mm^3

   AST (SGOT) less than 2.5 times the upper limit of normal (ULN)

   ALT (SGPT) less than 2.5 times ULN

   Total bilirubin less than 1.5 times ULN

   Creatinine <1.5 mg/dl

   β-HCG = negative

5. Karnofsky performance status ≥60
6. Signed informed consent

Exclusion Criteria: History of IPT, TB in the past 3 years, current TB, history of exposure to a person with known isoniazid resistant TB, history of hepatitis or active liver disease, current therapy with warfarin, phenytoin or carbamazepine, history of non-adherence to chronic therapies.

Study Medications

Isoniazid (dosed per weight according to TB Program Guidelines) daily for 6 months then placebo for 30 months<br>PLUS<br>Vitamin B6 25 mg per day continuous (36 months)

OR

Isoniazid (dosed per weight acc. to TB Program Guidelines) daily continuous (36 months)<br>PLUS<br>Vitamin B6 25 mg per day continuous (36 months)

1.0 BACKGROUND AND RATIONALE

1.1 Background Rationale for IPT

High TB case rates in sub-Saharan Africa have been largely attributed to the escalating human immunodeficiency virus (HIV) epidemic, presumably related primarily to endogenous reactivation in persons coinfected by both *Mycobacterium tuberculosis* and HIV.[1] The annual risk of developing active TB in HIV-positive individuals coinfected with *M. tuberculosis* is estimated to be 5-10% compared to a lifetime risk of 10% in HIV-negative individuals.[2-5]

Results from clinical trials in Uganda, Zambia, and Kenya, and a meta-analysis of four clinical trials in Haiti, Kenya, USA, and Uganda have been published demonstrating that isoniazid (INH) prevents TB in HIV-infected persons in settings of high TB prevalence.[6-9] The meta-analysis estimated an overall efficacy (1 - Risk Ratio) of 50% for all HIV-infected patients regardless of TST status, and 70% in those with a positive TST. The risk ratios for active TB in the INH treatment and placebo groups by tuberculin skin test (TST) status are summarized in Table 1.

Table 1. Efficacy of six months of INH for preventing active TB in HIV-infected patients by tuberculin skin test (TST) status.

Study	Country	Risk Ratio for active TB after IPT vs None			
		Mean yrs Follow-up	All pts	TST+	Anergic/TST-
Hawken 97	Kenya	1.8	1.0	0.60	1.2
Whalen 97	Uganda	1.1	--	0.33*	0.83
Mwinga 98	Zambia	1.8	0.59	0.25*	0.86
Wilkinson 98	(meta-analysis)	--	0.51*	0.32*	0.62

* 95% confidence interval does not include 1.0

These trials have also shown the safety of INH among HIV-infected persons. Side effects were rare, as illustrated by an only 0.6% drop-out rate among persons treated in the Uganda trial.[7] Ten to 20% of those receiving INH developed mild liver abnormalities, demonstrated by an elevated serum aspartate aminotransferase, which tended to resolve even if INH was continued. Peripheral neuropathy was another side effect of INH therapy; co-administration of pyridoxine (vitamin B6) has been shown to reduce its occurrence.[10]

Based on these encouraging results, UNAIDS issued guidelines in July 1998 recommending isoniazid TB preventive therapy (IPT) as a component of basic care for PLWH in high TB prevalence settings.[11]

1.2 Duration of IPT

A recent trial in Zambia demonstrated that the protective effect of limited IPT may not be prolonged.[12] TB case rates became indistinguishable between IPT-treated and placebo-treated patients 36 months after therapy. This has led to speculation that the 6-month regimen may be insufficient for conferring long-term protection against TB, either because *M. tuberculosis* reinfection occurs in settings with high TB prevalence or because six months of INH is inadequate for the complete sterilization that may be necessary to prevent the transition from infection to disease in advanced stages of immunodeficiency.

1.3 Rationale for IPT in Botswana

In 1999, approximately 29% of the population 15-49 years of age in Botswana was estimated to be living with HIV infection.[13] The TB case rate has more than doubled in Botswana during the 1990s (from 202/100,000 in 1989 to 521/100,000 in 1999) and is now among the highest rates in the world.[14] The strong association between HIV prevalence and the TB rate in Botswana is clear. Approximately 85% of hospitalized TB patients in Gaborone and Francistown during 1997-98,[15] and 73% of TB patients diagnosed in an outpatient setting in Gaborone during 1997-99 were also HIV-positive [16]. Preliminary results from an autopsy study conducted at Nyangabgwe Hospital in Francistown, Botswana, during 1997-1998 suggest that TB may account for 36% of deaths in adults dying of AIDS.[17]

Although no systematic studies have been performed, evidence suggests that the vast majority of adults in Botswana are asymptomatically infected with *M. tuberculosis*. Since TST may not be an accurate indicator of TB infection among HIV-positive patients, studies of HIV-negative adults in a population provide some indication of the

prevalence of underlying TB infection in the population, since the prevalence of infection would be expected to be similar regardless of HIV status. Tuberculin skin testing of contacts during an outbreak investigation of TB in a US government office in Botswana demonstrated that all 40 employees born in Botswana had induration (10 mm.[18] A high infection rate in early childhood also suggests that the underlying prevalence of TB infection in adults will be high. A community survey in Botswana found that 7% of children (median age 2 years) had a positive TST and that known exposure to TB was associated with a positive test.[19] In Zambia, 62% of HIV-negative patients attending an STD clinic in Lusaka in 1990 were found to have a positive TST (cut-off 10 mm induration).[20] Similarly, in Uganda, 58% of HIV-negative postpartum women studied from 1988-89 had a reaction (10 mm.[21] TB case rates in African countries heavily impacted by HIV have more than doubled during the 1990s since these skin test studies were performed, suggesting that the current prevalence of *M. tuberculosis* infection in adults may be even higher. Thus, a significant proportion of the adult population in Botswana is likely to be coinfected with HIV and *M. tuberculosis* and is at substantial risk of developing active TB over the next several years. In addition, INH is likely to be effective, since the rate of resistance was shown to be low in a recent nationwide survey.[22]

1.4 The IPT Pilot Program in Botswana

In March 2000, the Ministry of Health approved the pilot provision of IPT to eligible HIV-positive clients who are 1) identified at the VCT centers in Gaborone and Francistown which were established with CDC support under the new LIFE Initiative; 2) postpartum women enrolled in the MTCT pilot project; and 3) patients diagnosed in the clinical services. Following UNAIDS guidelines, the pilot IPT project offers six months of IPT, without the use of either routine liver function tests or tuberculin skin testing. PLWH

eligible for the pilot must be at least 18 years of age and live in the MTCT pilot areas of Francistown, Gaborone, and Ramotswa district. To exclude patients with active TB, patients are screened by history, physical exam, and chest radiographs (although radiographs may be discontinued if local operational research demonstrates that screening for signs and symptoms of TB alone is sufficient). Other exclusion criteria for the government pilot IPT program include active TB in the previous three years, advanced AIDS, active hepatitis, a history of non-compliance with other chronic therapies, or a history of intolerance to INH. The IPT algorithm and notes to the algorithm are included in Appendix 1.

To date, the government IPT program has screened 935 patients, of whom 471 were started on treatment.[23] The most common reasons for not starting patients on IPT included symptoms suspicious for active TB (237), loss to follow-up during the chest radiograph process (112), or abnormal chest radiograph (22). Of those who have started IPT, compliance has been over 50%. Four patients were discontinued because of symptoms consistent with active TB after beginning IPT, and are currently under evaluation for confirmation and INH resistance testing.

2.0 STUDY OBJECTIVES AND ENDPOINTS

2.1 Study Objectives. This study will test the hypothesis that, in a setting of high TB prevalence, continuous IPT (defined as taking INH daily throughout the study duration, up to 36 months) is superior to a six-month (limited) IPT regimen protecting against active TB. Specific objectives will include:

2.1.1 Determine the proportion of PLWH who are eligible for the government IPT pilot program who have a positive TST, abnormal chest radiograph, and active TB at the time of attempted study enrollment;

2.1.2 Estimate subject adherence with limited vs. continuous INH;

2.1.3 Determine if continuous IPT is superior to limited IPT to prevent active TB (definite, probable and possible) and death in PLWH;

2.1.4 Ascertain the causes of morbidity and mortality among subjects;

2.1.5 Quantify the generation of INH resistance in subjects who develop culture-positive TB during or after IPT;

2.1.6 Identify risk factors for the development of TB in persons taking limited vs continuous INH, including demographic characteristics, adherence, TB exposure, TST results, chest radiograph findings, CD4, and total lymphocyte counts).

2.1.7 Store and perform drug susceptibility testing and genetic analysis on *M. tuberculosis* isolates in subjects who develop culture-positive TB.

2.2 Study Endpoints. The primary endpoint will be active TB (pulmonary or extrapulmonary) occurring after the participant initiates coded medication and before the end of the 36 months of study medication. Secondary endpoints will be 1) the combined endpoint of definite and probable TB (possible TB being excluded), 2) the combined endpoint of active TB and deaths in which TB was not excluded as a cause, 3) the combined endpoint of active TB and deaths, 4) adverse events, and 5) death. Outcome variables will be CD4 cell count and PPD positivity. These variables may assist the development of future IPT algorithms, and in clinical management of subjects. Clinical progression of HIV disease will also be reported. When participants reach an endpoint other than death (and including completion of study medication), they will continue to be followed off study medication on a quarterly basis for the duration of the Study. Persons completing 36 months of study medication will continue to be followed for an average of 2.5 years as in the Zambia follow-up study [12]. This will provide important insights into the long-term effects of active tuberculosis in PLWH which are thought to

be grave even in the face of completion of anti-tuberculosis therapy as well as any possible TB outcomes among those who complete 36 months of the study medication. Cases of TB occurring after the 36-month period of study medication will not be included in the primary analysis. Given that in PLWH smear-negative and culture negative TB are common, a blinded independent assessment of patients' data may be necessary when insufficient or inconclusive endpoint data are available with the use of an endpoint committee.

3.0 STUDY DESIGN AND METHODS

3.1 Study Population and Eligibility

Participants must be HIV-positive, at least 18 years of age (the age cutoff in Botswana for being able to request a HIV test without parental permission), and consent to participation in the study. In addition, participants must have a Karnofsky performance score of ≥60 (indicating that they are in not in bed >50% of the day, do not require assistance for activities of daily living, and do not require frequent medical care [24]). Participants may be either TST-positive or -negative, and may be taking antiretroviral treatment.

Exclusion criteria will include active pulmonary or extrapulmonary TB (by positive AFB smear or culture), a history of TB in the last three years, a history of IPT, exposure to a person with known INH-resistant TB, active hepatitis or other liver disease (AST or ALT greater than or equal to 2.5 times ULN and/or total bilirubin greater than or equal to 1.5 times the upper limit of normal), current therapy with warfarin, carbamazepine or phenytoin, or a self-reported history of non-adherence with other chronic therapies. All enrollees will receive a baseline chest radiograph. A study physician may permit an individual to be included in the study with an abnormal chest radiograph in such situations where the abnormality is not consistent with active TB or reflects residual scarring from a history of documented treated TB over 3 years ago or documented treated pneumonia.

Children are excluded primarily at the request of the BNTP which is because the current Botswana IPT pilot program excludes children. The policy for exclusion was based on the fact that children are less likely to have acquired *M. tuberculosis* infection than adults since risk of *M. tuberculosis* infection is cumulative over time.

If a woman is found to be pregnant at the time of enrollment, she will be referred to the MTCT program and encouraged to return 6 weeks postpartum for reconsideration for enrollment. This is consistent with the national IPT program guidelines, in that pregnant women are not given IPT. If pregnancy occurs during trial follow-up, the woman will again be referred to MTCT, but will be continued in the trial.

After enrollment, criteria for withdrawal from the study will be the subject's voluntary withdrawal, death, or determination of ineligibility after enrollment. Subjects that develop medical complications of AIDS, are suspected to have had INH toxicity, or develop non-tuberculous disease will continue to be followed for the 36-month duration of the study. During the course of the study, death certificate records and electronic TB register data will be periodically examined for those that made permanent moves from Gaborone or Francistown and those who did not return for any follow-ups. Participants who are determined to be ineligible for the study may remain eligible for the government IPT program, and will be re-evaluated for such.

3.2 Study Design and Flow

Appendix 1 shows the algorithm for enrollment into the government IPT program. The staff at ten selected centers in Gaborone and Francistown will be instructed to screen PLWH, first using the published national guidelines (Appendix 1). If eligible for the

government IPT program, the potential participant will then be screened according to more stringent guidelines for enrollment into this study:

Study visit 1

1. The study will be explained and consent for participation obtained (Appendix 2)
2. An enrollment questionnaire (Appendix 3) will be administered
3. TST, chest radiograph, CD4 count, complete blood count, pregnancy test, and blood chemistry will be performed

Study visit 2 (day 2 or 3 after enrollment)

1. The chest radiograph, laboratory results, and TST will be read and results will be recorded on Appendix 4
2. If still considered eligible, subjects will then be randomized to receive six months of INH followed by placebo or to receive continuous INH (as described in 3.6). All subjects will receive pyridoxine to reduce the risk of peripheral neuropathy.
3. The study nurse, who is blinded to the result of randomization, will provide a one month supply of the study medications (a bottle containing 128 tablets of 100 mg isoniazid), and schedule the subject to return in two weeks. Subjects will be advised to return to the clinic if they develop any illnesses or symptoms while on medications, including but not limited to cough, fever, night sweats, nausea, or vomiting, pruritis, or rash.

Study visit 3 (after two weeks of the study medications)

1. The subject will be evaluated for adverse medication effects such as rash or jaundice, and have blood drawn for repeat liver function tests. Results will be recorded on Appendix 5.
2. An additional two-week supply of study medications will be provided

Study visit 4 (after four weeks of the study medications)

If there were no significant abnormalities on the blood drawn during study visit 3,

the subject will be given a one-month supply of medications and instructed to return once a month for follow-up

Study visits 5 - 8

The subject returns to the clinic monthly to be evaluated for active TB, side effects, adherence, and for a refill of study medications. Results of any evaluations will be recorded using Appendix 6.

Study visit 9 (after six months of the study medications)

1. Subjects who had been randomized to six months of INH will be switched to placebo in a blinded fashion, while subjects who had been randomized to continuous INH will continue receive INH.

2. Both groups will continue to receive pyridoxine, return to the clinic monthly to pick up medication refills, and attend study clinic visits every three months to be screened for side effects of INH, AIDS progression, TB symptoms and TB exposure. During other months a new bottle of study medications will be provided, and participants will have a more detailed interview only if they report complaints. Furthermore, every six months a participant acceptability form will be completed to ensure that the participant is able to follow the medication regimen, and continues to comprehend the goals of the trial. Participants still in the study but off study medications (e.g., those who suffered a prolonged illness or adverse symptoms requiring discontinuation of study drugs for more than 4 weeks) will be followed every three months.

3.3 Follow-up

Study visit 5 (after two months of the study medications) is the first of the routine follow-up study clinic visits. All subjects will be followed monthly while taking study medication and quarterly if no longer taking study medication for 36 months. If a study participant has not reached a study endpoint by the 36th month, she/he will be invited to return for quarterly visits

until the last participant enrolled in the trial takes her/his last dose of study medication (this is when contact with all participants will cease and new data collection ends). During these follow-up visits, the subject will be evaluated for active TB, clinical progression of HIV disease, other adverse events including drug toxicity, and adherence. Results will be recorded using Appendix 6. Unless contraindications are identified, subjects will be given a one-month supply of medications from the study clinic at each follow-up visit. Subjects will also receive safe sex counseling at each visit. Due to the recent Botswana government policy of free anti-retrovirals, should a subject meet criteria for anti-retroviral therapy, he/she will be referred to the Ministry of Health’s ARV clinic services.

3.3.1 Active tuberculosis. Screening for the development of active TB will be done with a symptom screen (Appendix 6). If the symptom screen is positive, the subject will be told to halt INH until active TB is excluded. The subject will then undergo chest radiography, and AFB smear and culture using a liquid culture system. Active TB includes definite, probable and possible TB. “Definite TB” is when one or more cultures are positive for TB or there are two positive sputum smears and clinical evidence consistent with TB. “Probable TB” is when one or more smears are positive or there is histology suggestive of TB (e.g., granulomata) with clinical evidence consistent with TB. “Possible TB” is when there is absent or negative smear or histology and absent or negative culture for TB and clinical evidence consistent with TB including a clinical response to anti-TB therapy. A “false positive” is when there is one culture positive for TB with clinical evidence inconsistent with TB. “Unlikely TB” is when there is a case not falling under any of the above four categories (i.e., not definite, probable, possible or false positive)..

3.3.2 Clinical progression of HIV disease: Clinical progression is defined as the first occurrence of an acquired immunodeficiency syndrome (AIDS)-defining condition [25]

or as a recurrence of PCP, esophageal candidiasis, herpes simplex infection, disseminated herpes zoster, or septicemia due to non-typhoidal salmonella.

3.3.3 Drug toxicity. Drug toxicity will be evaluated by the participating physician and graded by criteria listed in the AIDS Clinical Trials Group (ACTG) Table for Grading Severity of Adverse Outcomes in Adults (see Appendix 7). The protocol for recognizing adverse events will be facilitated by a checklist incorporated in the data forms present in the subject's chart. Physicians will also use their discretion to order additional tests, as necessary.

For Grade 1 toxicity: if laboratory abnormalities are present, tests will be repeated one week after the abnormal values were noted. Medication may be continued.

For Grade 2 toxicity: If laboratory abnormalities are present, tests will be repeated every 2-3 days. Medication may be continued. If subjects develop persistent (i.e., more than two months') hepatic enzyme elevations, they will continue on medication and be monitored by symptoms and quarterly liver function tests.

For any Grade 3 toxicity, or Grade 4 toxicity for hemoglobin: Medication will be discontinued. The subject will be monitored and receive organ-specific supportive care as his/her condition warrants.

All serious, unexpected, and at least possibly related to study interventions adverse events will be promptly reported to the Botswana and CDC ethics committees. At the time of annual continuing review of the protocol, a summary listing of all adverse events (including anticipated) will be reported to both committees. All adverse events will be reported to the Data and Safety Monitoring Board.

Given the high estimated prevalence of chronic hepatitis B infection in sub-Saharan Africa, the greater risk of death from liver disease among persons co-infected with hepatitis viruses[34] and

HIV and the implication of continuous IPT, all persons who develop grade 2 or higher liver function abnormalities will be tested for evidence of active hepatitis B and hepatitis C infections. To determine whether there is a greater risk for hepatitis among these persons as compared to those who do not develop grade 2 or higher liver function tests, two participants without evidence of hepatic enzyme elevations but with a similar medication profile will be selected as controls for each case.

3.3.4 Adherence. A subject will be encouraged at enrollment and at each follow-up to take all medications as prescribed. In order to encourage adherence, subjects will be asked to bring any remaining medication to every follow-up visit. Pill counts will be done, results recorded (Appendix 6), and reasons for discrepancies will be explored. Results of pill counts will be recorded, but the subject will not be removed from the study even if non-adherence is suspected.

“Enablers” will be provided to the study participants in order to maintain their willingness to continue to participate. The enablers will include cash (30 Pulas = US$5) for each visit as compensation for transport to and from the clinics. Other enablers such as caps, T-shirts and participant mini-workshops for coping with HIV/AIDS will be provided at significant milestones in the study. Additional reimbursements to participants will be provided for unscheduled, required study-related visits for such instances as when a blood sample was lost or clotted.

In order to estimate overall adherence, annually a random selection of 20% of study participants will be consented to provide urine samples to assay for INH metabolites. The samples will be sent to a laboratory unaffiliated with the study. The laboratory will report the aggregate number of positive samples without revealing participant identification numbers so that study personnel

will remain blinded as to whether a certain study participant is taking the placebo or the active investigational agent.

A case control analysis will be conducted anonymously to identify factors associated with non-adherence among study participants. More specifically, the objectives will be to determine differences in knowledge, attitudes and beliefs between the cases and controls and reasons for stopping study medication. Cases will be participants who stopped taking the study medication yet agreed to continue keeping quarterly appointments as well as persons who were lost to follow up. Controls will be those who continued to take the study medication. The study will be conducted in two stages: 1) formative research (focus groups, key informant interviews, and cognitive interviews) and 2) a survey study. This assessment will be conducted by a contracted third party in order to enhance openness by study participants. This degree of openness may not occur if study participants were interviewed by study nurses whom they know. Cases will include any participant meeting the above-stated criteria and 2 controls will be interviewed for each case interviewed. A reimbursement of P60 (~$10) will be provided to those participating in these interviews as they may take up to 2 hours.

3.3.5 Loss to follow-up. If a subject is late for a follow-up visit, study nurses will attempt to locate the subject by calling or visiting the subject's home, and will record the reason for loss to follow-up. During the first six months of open label INH administration, if a study participant has been off study medication for 30 consecutive days or for two 14 day periods s/he will no longer receive study medication but will continue to be followed on a quarterly basis (every three months). Once a study subject is on coded medication (after month 6), s/he may, while urged to be adherent to the treatment, return on coded medication at any time even if non-adherent for a prolonged period. A standard symptom questionnaire - using the same criteria as the screening visit - will be administered including a chest radiograph and hepatic enzyme tests in order to

avoid resuming IPT in a person with active TB or active hepatic disease. Study medications will end at the 36th month after enrolment. With the exception of study subjects who decline participation in the trial, die, develop tuberculosis, or were found to be ineligible after enrollment, all subjects will be followed for 36 months regardless of whether they take the study medications.

For those subjects who are not located, the Electronic TB Register and death certificate review will be conducted at periodically during the course of the study. For those subjects who are hospitalized, chart review will be conducted. For those subjects who died, a verbal autopsy will be conducted by the study physician (Appendix 8).

3.3.6 Death. A significant effort will be made to determine the cause of death for all enrolled subjects. In order to improve retention in the study as well as the promptness of death notifications and determining the cause of death, P1,000 (=$167) will be given as an enabler to family members in the form of a contribution towards the cost of a participant's funeral. Three sources of information will be utilized to determine the likelihood that a subject expired from TB: the opinion of the clinician responsible for the subject's care, a verbal autopsy questionnaire will be administered to a household relative of the deceased, detailing symptoms pertinent to the likely cause of death (Appendix 8), and a necropsy will be sought. Death of a subject will be classified according to the following
scheme:

Definite TB death - clinical and/or verbal autopsy evidence consistent with TB as the primary cause of death and a positive culture for *Mycobacterium tuberculosis* from any organ or body fluid at necropsy

Probable TB death - clinical and/or verbal autopsy evidence consistent with TB as the primary cause of death and a positive smear for AFB or granulomata on pathological examination of any organ or body fluid necropsy

Possible TB death - Death in a subject with clinical or verbal autopsy evidence consistent with TB as the primary cause of death and a determination that any clinical or verbal autopsy or necropsy evidence suggesting the presence of a concomitant diagnosis at death is not deemed to be the primary cause. Additionally a deceased individual having one or more cultures positive for TB and clinical evidence inconsistent with TB should be categorized as a possible TB death.

Unlikely TB death - death in a subject with necropsy or clinical or verbal autopsy evidence suggesting a primary cause of death other than TB

Unknown TB death - death in a subject with no clinical information or verbal autopsy, or where the information available does not suggest any specific cause of death

3.4 Medication Supply, Administration, and Dosage

3.4.1 Supply. INH, placebo and pyridoxine tablets will be manufactured by West-ward Pharmaceutical Corp. (Eatontown, New Jersey), distributed by Versa Pharm, Inc. (Marietta, Georgia) and labeled and shipped by the U.S. Department of Health & Human Services' Program Support Center (PSC) (Perry Point, Maryland). Tuberculin Purified Protein Derivative (PPD) skin tests will be supplied as sterile solutions of 2 TU of strain RT-23 per 0.1 ml in 1.5-ml vials, for intracutaneous use, manufactured by Statens Serum Institut, Copenhagen, Denmark (distributed by Biovac, South Africa).

3.4.2 Administration. One month supplies of medication will be picked up at the study

sites and self-administered by the subject. Uniform efforts will be made to ensure adherence (see below).

3.4.3 Dosage. Subjects in the cohort study will receive the same dosage of both INH and pyridoxine as taken during the government IPT program. The IPT Study will follow the National TB Program guidelines and routine government IPT program for INH:

100-200 mg if the subject weighs <30 kg

300 mg if the subject weighs ≥ 30 kg

The IPT Study will stock INH in 100 mg scored tablets. Nearly all subjects in the study will weigh at least 60 kg and will take three tablets daily. Should a subject weigh less than 30 kg, the dosage will be based upon a rule of 5 mg/kg. Subjects will also be provided pyridoxine, 25 mg daily. Subjects randomized to receive placebo will receive the same number of pyridoxine tablets plus tablets identical in appearance and number to INH.

3.5 Laboratory and Diagnostic Testing

3.5.1 Mycobacteriology: AFB smears will be performed according to the routine of the Lancet Laboratory located in Johannesburg, South Africa. Mycobacterial cultures will be performed at Lancet Laboratories using liquid media (MGIT, Becton-Dickinson, Sparksville, MD).
Sputum specimens for mycobacteria will undergo digestion, decontamination, and centrifugation, and the sediment will be used for AFB or fluorescent smear and culture inoculation. Cultures will be incubated for up to six weeks to observe for growth. Specimens from any subject who develops active TB will undergo INH susceptibility testing using the MGIT system according to published manufacturer guidelines.

3.5.2 Chest radiography: Chest radiographs are required at enrollment and anytime the subject offers symptoms consistent with TB. These will be done on-site at the study clinic, and will be interpreted by a radiologist who will only know that the film was ordered for "rule-out TB" (not as part of a research study).

3.5.3 Tuberculin skin testing: A positive TST is defined as ≥5 mm induration to 2 TU of PPD using the Mantoux method [28]. The optimal time for reading the TST is 48-72 hours after PPD administration; however, if a subject's reaction is read beyond 72 hours and is >5 mm, this will be considered positive. BCG vaccination is given as a single dose to more than 90% of children at birth in Botswana,[14] but reports suggest its affect on TST reactivity is variable and wanes over time.[29] Therefore, we will use standard TST interpretation criteria in the determination of a positive TST.[28]

3.5.4 HIV and CD4 cell count testing: The HIV test that is required for enrollment into the government IPT pilot program will be performed according to the guidelines and procedures currently approved by the Botswana Ministry of Health. Sera will be evaluated by two ELISAs run in parallel (Ortho Kit HIV-1 and HIV-2 or Abbot Murex) or dual rapid ELISA (Determine Assay, Unigold, Capillus, or Orasure). Two positive ELISA tests will be considered positive for HIV infection. Two negative ELISA tests will be considered negative for HIV infection. Discordant results will be confirmed by a third ELISA. Enumeration of CD4+ T-lymphocyte cell counts and CD8+ T-lymphocyte cell counts will be performed in Botswana by FACSCount (Becton Dickinson, San Jose, CA, USA). After enrolment, a rapid serologic test for HIV will be repeated in all subjects with a current CD4 lymphocyte count over 500 cells/mm^3 and in a random sample of 10% of subjects with fewer than two CD4 counts below 500 cells/mm^3 in order to confirm that they are indeed HIV infected.

3.5.5 Blood tests. Hemoglobin, hematocrit, platelet, and white blood cell count with differential will be done with the Coulter counter (Coulter Diagnostics). Liver function tests will be done according to the clinic routine.

3.5.6 Drug Susceptibility Testing (DST).

All culture positive TB isolates will be tested for sensitivity to isoniazid, rifampicin, ethambutol, streptomycin. Isoniazid susceptibility testing is routinely performed at both low (0.2 μ/L) and a high (1.0 μ/L) levels of drug concentration.

3.5.7 Genetic Analysis of *M. tuberculosis* Isolates

Spoligotyping, Mycobacterial Interspersed Repetitive Unit Typing (MIRU) and/or Restriction Fragment Length Polymorphism (RFLP) using IS6110 fingerprint testing will be conducted on any *M. tuberculosis* isolates according to standard methods at the CDC's Atlanta facility.[30] The purpose of this is to identify clusters within the IPT facilities which suggest recent transmission, as well as to estimate the relative burden of recent transmission compared with reactivation of latent *M. tuberculosis* infection. This information will assist TB Control Programme efforts. The presence of mutations of *katG* and *inhA* will be investigated on these specimens as mutations in these genes have been associated with isoniazid resistant MTB.

3.6 Randomization

Randomization schedules will be prepared to assure an allocation ratio of 1:1 (limited or continuous INH). Randomization of drug and placebo assignment will be achieved by using the permuted-block randomization method in which one of ten 5-digit codes (to which study clinicians will be blinded) will be pre-assigned to all potential patient identification numbers by clinic site prior to the launch of the study. Each of the proposed 10 study clinic sites will have a table that lists the patient identification number and the corresponding, randomly assigned 5-

digit study drug code. The key to the code will be kept at CDC in Atlanta and at PSC in Perry Point, MD.

3.7 Interim Analysis

Safety and efficacy data will be reviewed by an independent Data and Safety Monitoring Board (DSMB). The DSMB will be comprised of four members who are not involved with enrolling participants into the trial. The DSMB will be permitted to seek additional expertise, including pharmaceutical representatives (although these will not be voting members). The following persons have given their consent for participation on the DSMB:

1. Karin Weyer, Dsc: Director, TB Operational and Policy Research Unit, Medical Research Council, South Africa. Dr. Weyer has published over 100 articles, book chapters and manuals on tuberculosis in Africa, and has made numerous presentations at conferences during 21 years of TB research. She serves as a permanent consultant on Tuberculosis Control to the World Health Organization.
2. Andrew Nunn, BS MSc, Head of Division Without Portfolio, British Medical Research Council Clinical Trials Division Unit, and Honorary Senior Lecturer at the University College London Medical School. He is a statistician by training and has published numerous articles in peer reviewed journals including papers on tuberculosis in a number of countries in Africa over the last 30 years.
3. Ndwapi Ndwapi, MD, Director of MASA National ARV Programme (Gaborone, Botswana) which provides anti-retrovirals to a population of tens of thousands of HIV-infected patients. He is a senior instructor of the KITSO national training program for anti-retroviral therapy and care for HIV/AIDS patients. He is presently involved in research projects addressing basic science as well as clinical care of HIV-infected individuals.

4. Jonathan Levin, PhD, Senior Statistician, Medical Research Council, South Africa. He has contributed to the publication of a wide range of medical issues in South Africa, including such clinical trials as a WHO-sponsored TB/HAART study in Tanzania and Uganda.

Data monitoring will use the Lan-DeMets method as a guideline for early stopping. A single interim analysis will be scheduled for 24 months from the start of enrollment. This time was selected because half of subjects will average 18 months of follow-up, which is the time to which some trials have shown that the protective effect of INH becomes indistinguishable from that of placebo [12]. However, if the incidence of adverse events is noted to be high, an earlier interim analysis may occur before the scheduled 24 month analysis. The DMSB will receive trial results from the principle investigator (TS), who will not communicate interim results to any other investigator. The primary criterion for stopping the trial before the scheduled completion will include sufficient primary endpoints (active TB) which demonstrate a difference between the 6-month and continuous arm of IPT that is significant at the $P<0.001$ level. The DSMB might also advise modifying the trial under several circumstances:

1) If INH resistance is detected to be ≥10% (twice 1999 national levels) in subjects who develop culture-positive TB, the DSMB may recommend early discontinuation of trial at the request of the Botswana ethical review board (the Health Research and Development Committee, HRDC);

2) If the incidence of adverse events is noted to be high;

3) If it becomes apparent that the sample size will not be reached within the allotted time, the DSMB may recommend to modify enrollment criteria, to extend the enrollment period, or to halt the trial.

If it is considered necessary to halt the trial early, the enrolling clinicians will be promptly notified by the principle investigator by phone and FAX. If the continuous

INH arm is found to be superior to six months, subjects receiving INH or placebo will be informed and offered continuous INH.

4.0 SAMPLE SIZE ESTIMATES

Assumptions:

1. Subjects will be equally allocated to limited and continuous INH.
2. Most PLWH in Botswana are infected with *M. tuberculosis*, although HIV-infected persons may be anergic to tuberculin skin testing.
3. Among HIV/TST-positive persons, continuous IPT will decrease the rate of TB from that seen after six months of IPT by 50% (from 3.0 to 1.5 per 100 person-years, or from 4.0 to 2.0 per 100 person-years).
4. Up to 21% of subjects may be unable to take INH or be lost to follow-up; this proportion should not vary significantly between study treatment groups.

Using EpiInfo 6.04 (CDC, Atlanta GA) "sample size function, cohort or cross-sectional study" with confidence level of 95%, power of 80%, and a frequency of disease of 10% among those in the placebo arm (i.e., 6 months isoniazid followed by placebo or “unexposed”), if 6 month IPT results in a rate of 3.0, the study requires 474 subjects per arm. Based upon the observed attrition rate in the trial by December 2005 (21%), and because stratification will be done based on TST status (positive, (5mm, and negative, <5mm) and CD4 counts ((200 cells/mm3 and >200 cells/mm3) we assume that 60% will have TST (5mm), the estimated sample size was inflated and rounded up to 1,000 in each arm.

5.0 DATA COLLECTION, ANALYSIS AND MANAGEMENT

Clindex software (Fortress Medical Systems, Inc.) will be used to store information received from case report forms. Only the patient’s study identification code and no personal identifiers

will be kept in this database. Access software (Microsoft, Inc.) will be used to store personal identifiers. The latter database will be accessible only to clinicians who may contact the study population.The data will be double entered into a password-protected database. Discrepancies in entry will be resolved by the study nurses and data clerks. Informed consents with both names and the unique identifier will be stored in locked cabinets in the BOTUSA office in Gaborone and destroyed after data analysis is complete. Only clinicians involved in patient care and the principle investigator (TS) will have access to this cabinet during the study. No other employees of BOTUSA, the CDC, or the Ministry of Health will be able to link clinical or scientific data to any subject. Final authority over the data rests with BOTUSA and the Botswana Ministry of Health.

The analysis will be primarily conducted by the principle investigators. There is statistical support available at the CDC headquarters in Atlanta in the person of Dr. Nong Shang (CHSRB, DTBE).

The treatment groups will be compared with chi-square tests or Fisher's exact tests for categorial variables, and with Students-t tests and/or Wilcoxon rank sum tests for continuous variables. Time-to-event analyses will be performed with proportional-hazards regression. The proportion assumption will be checked. Both unadjusted and adjusted analyses will be performed to estimate odds ratios for the incidence of TB, mortality, and progression of HIV disease. Primary analyses will be performed on an intention-to-treat basis. All randomized participants (i.e. those starting coded medication) will be included in the intention-to-treat analysis, including those who missed substantial amounts of treatment or had been given the wrong treatment. All P-values will be two-sided. For dichotomous variables, relative risks with 95% confidence intervals (CI) will be calculated. Step-

wise logistic regression will be used to evaluate variables found to be significant in the univariate analyses using SAS (SAS Institute Inc., Cary, NC), a statistical software package.

6.0 DEVELOPMENT OF MATERIALS FOR INFORMATION, EDUCATION, AND COMMUNICATION

The overall goals for the development of information, education and communication (IEC) materials is to increase awareness about the study among health care workers, nongovernmental organizations (NGOs), HIV Voluntary Counseling and Testing Centers (VCTs), potential study participants, and to recruit participants into the study. A systematic health education planning process will be used to develop the IEC materials based upon well-established models [32]. The steps in this process include 1) strategy formulation; 2) channel and material selection; 3) material development, including formative evaluation; and 4) implementation.

6.1 Strategy formulation

Between March 10-20, 2003, a needs assessment was conducted with key informants in order to: (1) notify key informants about the study and determine their willingness to assist BOTUSA with participant recruitment; (2) determine methods for educating and recruiting study participants; (3) determine methods for educating health care providers at the government clinics and counselors at NGOs about the study and for assisting with participant recruitment; (4) determine methods for ensuring participant adherence.

6.2 Channel and material selection

Based on the needs assessment results, the following draft materials will be developed:

- Buttons to be used to initiate questions and discussion about the study. The target audience are clinic nurses and BOTUSA study staff.

- Posters will be placed in clinics and NGO waiting areas to increase awareness about the study. The target audience is potential participants, clinic, NGO, and VCT staff.
- A pamphlet that will contain condensed information about the study, eligibility requirements, and contact information. The target audience is potential study participants.
- A booklet that will contain detailed information about participation in the study, including information about transportation issues, monthly clinic visits, and adherence issues.
- The target audience is study participants.
- A calendar/reminder card that will be used by participants to assist with adherence to treatment and monthly monitoring clinic visits. The target audience is study participants.
- Two fact sheets: one targeted at clinic nurses and will contain information about the study, referral procedures, and contact information; the other targeted at NGOs, VCTs and will contain information about the study, referral procedures, and contact information.
- A staff schedule that is targeted at clinic nurses and will provide information about the BOTUSA staff schedule at the study clinic.

6.3 Material development (formative evaluation)

The draft IEC materials will be developed based on the needs assessment results and feedback from the IPT study coordinators. Formative evaluation will be utilized to ascertain the opinions, reactions, and beliefs of the target audience regarding: acceptability, feasibility, usability, readability, and comprehension of the IEC materials. The formative evaluation will address content, format, and design issues. The formative evaluation will use two approaches: focus groups and interviews.

6.3.1 Focus groups

Two focus groups will be conducted in each of the study sites – Gaborone and Francistown (total of 4 focus groups). Participants will be recruited from the target audience by BOTUSA study staff using standardized recruitment procedures. Each focus group will consist of 8-10 participants from the target audience. The focus groups will be conducted by a moderator from BOTUSA and the Botswana National TB Program. The focus group moderator will conduct the focus groups using a standardized guide (script) translated into Setswana

6.3.2 Interviews

Ten interviews will be conducted with health care workers and counselors regarding the content, format, and design of the fact sheets. The interviews will be conducted by a moderator from BOTUSA and the Botswana National TB Program.

Both the focus groups and the interviews will be conducted in Setswana or English and tape-recorded. The recordings will be transcribed and translated into English for analysis by CDC. Information from the interviews will be analyzed in aggregate form by CDC. No identifying information will be collected.

Assurance will be given to participants that the information discussed in the focus group or interview will be kept confidential, anonymous, and that their participation is voluntary – they may withdraw at any time. At no time during the data collection process will participants be asked to provide personal, or unique, identifying information (i.e., information such as birth date, social security number, first and last names address, etc.). The data collected will be reported on an aggregate basis – not an individual level – and all summary information will be provided to CDC in a manner that prohibits identification of individual participants.

6.4 Implementation

The IEC materials will be finalized based on the results of formative evaluation conducted with the target audiences and will be submitted to CDC IRB for approval. Final products will be available in English and Setswana.

7.0 PROTECTION OF HUMAN SUBJECTS

This protocol will be sent for ethical approval to the Institutional Review Board of the Centers for Disease Control and Prevention (U.S.A.), and the Health Research and Development Committee (Botswana).

7.1 Consent The consent form was developed in English in collaboration with Botswana Ministry of Health counterparts. The English version was determined to be at a Flesch Reading Ease of 72.5% and a Flesch-Kincaid Grade Level Score of 8 (using Microsoft Word tool). The consent form was then translated into Setswana and Ikalanga, and back translated to English to ensure consistency. Consent for participation in the study will be obtained in the subjects' native language. Study staff members are available to consent subjects in either English, Setswana, or Ikalanga. Literacy in Botswana, last formally assessed in 1993, was found to be just under 70%. However, it is thought that few young adults are illiterate given the relatively recent expansion of the education system.[31] Consent will be read to any potential subject if illiterate and consent will be by witnessed "X" on the consent form.

Pilot testing of the consent will be conducted by administering the consent to 4 members of our staff who are similar to those who are to enrolled, and then administering a questionnaire to test comprehension (Appendix 9). The questionnaire

will include 20 true-false questions. We will continue to develop the consent and pilot test it with 4 (new) members of our staff until the pilot subjects are able to answer 80% correct. In order to ensure that study participants continue to understand the objectives of the study and what it means to take coded medication, the comprehension will be re-administered every six months. If subjects fail the test they will not be terminated from the study but the study nurses will review issues that were not fully understood.

All potential participants are first asked a number of questions parallel to those required for entry into the National IPT Programme. For this phase, which includes a physical examination and a blood draw, a verbal consent is obtained. Until the results of the blood studies and chest radiograph return, the participant is given written materials related to the Trial as well as a copy of the consent form. When the potential participant returns and is found to meet eligibility criteria, a full written consent is obtained from the study participant.

7.2 Risks and Methods to Minimize Risk The physical risks of participation in the study include side effects from the study medication, INH. The incidence of adverse reactions to INH has been estimated to be 5.4 percent. Rash, fever, jaundice, and peripheral neuritis are the most prominent adverse reactions. Concurrent administration of pyridoxine (vitamin B6) will prevent INH-related peripheral neuropathy as well as nearly all other nervous system disorders attributable to INH. [26, 27]

Other adverse reactions include agranulocytosis, thrombocytopenia, vasculitis, arthritic symptoms, convulsions, mental abnormalities, xerostomia, epigastric distress, methemoglobinemia, tinnitus, and urinary retention. Severe and sometimes fatal hepatitis may develop after many months of treatment. The risk of hepatitis varies with age, ranging from 0.3 percent for patients 20 to 34 years old to 3 percent in those over 50. Symptoms appear 50 percent of the time within the first 3 months of therapy. The

fatality rate is about 10 percent, with poorer prognosis associated with an onset of symptoms after more than 2 months of therapy. [26, 27] At follow-up, research staff will attempt to detect any of these side effects early by measuring liver transaminases two weeks after IPT is initiated, and then by symptom screening. Study physicians may decide to perform liver function tests to diagnose INH-induced hepatitis.

Metabolism of phenytoin or carbamazepine may be impaired by INH, resulting in toxicity and isoniazid may slow the metabolism of warfarin. Absorption of INH is impaired by antacids that contain aluminum or antidiarrheals that contain kaolin. Therefore, concurrent medications that a subject may be taking are noted on the enrollment form so that the medical officer can make appropriate interventions to avoid medication interactions, and patients taking warfarin, carbamazepine or phenytoin therapy will be excluded from the study.

Other physical risks include those of the blood draw, which will be minimized by sterile technique, and the chest x ray, which delivers a negligible radiation dose.

The most important social and psychological risk is that of having one's positive HIV status inadvertently disclosed. Stigma for HIV is still quite severe in Botswana, and a subject might be rejected from his/her home, have employment terminated, or be ostracized. BOTUSA Project staff are already sensitized to the issues of participant confidentiality, and during training in preparation for the study, confidentiality will be additionally emphasized. However, it should be noted that it is widely-known that the national IPT program is available only to HIV-positive persons. By choosing to attend the IPT clinic, participants have already accepted some risk of disclosure, whether or not they are enrolled into the study. Within the national IPT program, participants attend the clinic monthly (similar to the study), and are supposed to be followed at their homes if they do

not return for follow-up (also similar to the study). It should be noted that follow-up within the national program is erratic, depending on the available human resources within an individual clinic (personal communication, Dr. Ruth Mwansa, former Director BNTP).

Methods to maintain data confidentiality are detailed above in the section, "Data collection, analysis and management" (5.0), and include the use of unique study identification number as the only personal identifier in the computerized database, a password-protected database, locked cabinets for participant records for which only the data manager and the principle investigator have access and participant identifiers to which only clinicians caring for patients, the data manager and the principle investigator have access.

The subject also incurs the inconvenience of participating in a long-term study, and traveling for follow-up visits. As mentioned in section 3.3.4, enablers will be provided including the cost of transportation to and from the clinics. In addition, the BOTUSA Project employs drivers and has vehicles that can be used when a subject needs transport not convenient through the public transport system.

7.3 Anticipated Benefits and Methods to Maximize Benefit

The results of this investigation will potentially benefit the subject, the AIDS Control Programme in Botswana, and PLWH in other settings with a high prevalence of TB. Benefits to the subject include screening for pregnancy, allowing for prompt referral to the MTCT program. The INH will reduce the subjects' risk of TB. The initial enrollment investigations and the follow-up will enable early identification and management of medical conditions. Subjects enrolled in the study will also have immediate access to any other treatments that become standard of care in Botswana (such as cotrimoxazole

and antiretroviral therapy).

7.4 Community Advisory Board

A community advisory board will be established in Gaborone and in Francistown. The membership will consist of approximately 10 individuals drawn from members of local and traditional government, advocacy organizations for people living with HIV/AIDS, community health professionals, social welfare institutions, academia, and the media. Community advisory boards are necessary for this trial in order to: enhance the acceptability and ethical propriety of the IPT trial; build community awareness of the IPT trial through its membership drawn from a broad spectrum of organizations in the community; identify and address community concerns regarding the IPT trial; build communication networks to support the IPT trial and report on progress of the trial to the wider community.

7.5 Participant Advisory Group

A participant advisory group (PAG) will be established in Gaborone and in Francistown. The membership will consist of approximately 10 individuals drawn from a diverse representation of all participating subjects. The PAG will be convened to enhance the quality, cultural appropriateness, and acceptability of IPT Trial procedures for study participants. The PAG will advise study investigators about recruitment venues and materials, methods and materials used to obtain fully informed consent, the conduct of study procedures, and the dissemination of study results. Study personnel will communicate with the PAG progress being made in the study.

8.0 FUNDING AND RESOURCES

BOTUSA will cover the costs incurred as part of research study, such as the cost of the research medications, liver function and pregnancy tests, the CD4 count enumeration,

tuberculin skin testing instruction and equipment, computers and programmers for data management, transport of specimens and/or subjects, and will provide personnel necessary for on-site implementation and management of the study: two nurses, one doctor (time), and one laboratory technician in each city (Gaborone and Francistown). BOTUSA will also provide full-time epidemiologist support.

The Government of Botswana will provide the VCT and MTCT clinic staff and facilities, INH and other TB drugs, HIV counseling and testing, chest radiography, the TB laboratory space and equipment, access to and maintenance for the National Electronic TB Register, and will pay for routine services to the subject, such as evaluation and treatment for opportunistic infections. In the event of any injury to a study participant, she/he will receive the standard of medical care available to all citizens of Botswana.

9.0 TIMELINE

EVENT	Time interval (from July 02)
BOTUSA training	2 weeks
Subject enrollment	12 months
Follow-up complete	36 months
Data cleaning	6 months
Analysis & writing	6 months

10.0 INVESTIGATORS AND RESPONSIBILITIES

The investigators have given verbal consent for submission of this protocol and report no conflicts of interest.

A. The BOTUSA TB Project

U.S. Embassy Gaborone

2170 Gaborone Place

Dulles VA 20189-2170

Phone: +267-3901-696

Fax: +267-3973-117

1. Taraz Samandari, MD PhD

   Email samandarit@bw.cdc.gov

2. Barudi Mosimaneotsile, RN, MPH

   Email mosimaneotsileb@bw.cdc.org

3. Samba Nyirenda MD

   Email nyirendas@bw.cdc.org

4. Tefera bw.cdc.org Agizew MD

   Email agizewt@bw.cdc.org

5. Zegabriel Tedla MD

   Email tedlaz@bw.cdc.org

6. Thabisa Sibanda MD

   Email sibandat@bw.cdc.org

Dr. Samandari will serve as principal investigator, will serve as point of contact for the DSMB, provide data for the interim analysis, and will also participate in manuscript preparation at all stages. Drs. Wells, Talbot, Nyirenda , Samandari and Ms. Mosimaneotsile contributed to study design. Drs. Wells, Samandari, Nyirenda and Kilmarx , and Ms. Mosimaneotsile will contribute to enrollment, analysis, and interpretation. Ms. Mosimaneotsile and Ms. Mathebula will coordinate participant enrollment, participant follow-up, and facilitate data entry in the southern part and the northern part of the country, respectively. Dr.s Nyirenda, Agizew, Tedla, Sibanda and Samandari will make clinical assessments on study participants for the diagnosis of TB, review causes of death and assess possible adverse events. All the above investigators will contribute to the preparation of the final manuscript.

B. Centers for Disease Control and Prevention

1600 Clifton Road, Mailstop E-10

Atlanta GA 30333

Phone: 404-639-8485

FAX: 404-639-1156

1. Lorna Bozeman MS

   Email llb1@CDC.GOV

2. Charles Wells MD

   Email charles.wells@otsuka.com

3. Peter H. Kilmarx MD

   Email pbk4@cdc.gov

4. Deborah A. Gust PhD MPH

   Email dgg6@cdc.gov

Ms. Bozeman, Dr.s Wells, Kilmarx, and Gust, contributed to study design, data analysis, interpretation, and final manuscript preparation. Dr. Wells left the CDC in June 2007 for Otsuka Pharmaceutical Corporation but remains a study investigator.

C. Ministry of Health

1. Themba L. Moeti MB BS, MSc

   Managing Director

   Africa Comprehensive HIV/AIDS Partnership

   Private Bag X033

   Gaborone

   Tel: 393-3842

Email: tmoeti@achap.org

2. Julius Mboya, MD MSc

Epidemiology and Disease Control Unit

Community Health Services Division

Private Bag 00269

Gaborone, Botswana

Phone: 267-391-0619

Fax: 267-390-2092

Email: jmboya@gov.bw

Dr. Mboya will serve as co-principal investigator. Both Drs. Mwansa (former Director BNTP) and Moeti (former Director BNTP) have had input into study design. Dr. Mboya will supervise the collaboration and communication between the CDC and the Ministry of Health. He will also review and contribute to the final manuscript.

D. Hospital Staff

1. Howard Moffat MBCHB, FRCP

Princess Marina Hospital

Gaborone, Botswana

Phone: 267-395-6730

FAX: 267-395-6354

Email: hjmoffat@info.bw

Dr. Moffat has contributed to protocol development and will assist in the interpretation of results. He will be involved in the preparation of the final manuscript.

11.0 REFERENCES

1. Cantwell M, Binkin N. Impact of HIV on tuberculosis in sub-Saharan Africa: A regional

perspective. Int J Tuberc Lung Dis 1997; 1: 205-14.

2. Selwyn PA, Hartel D, Lewis VA, et al. A prospective study of the risk of tuberculosis among intravenous drug users with human immunodeficiency virus infection. N Engl J Med 1989; 320: 545-50.
3. Allen S, Batungwananayo J, Kerlikowske K, et al. Two-year incidence of tuberculosis in cohorts of HIV-infected and uninfected urban Rwandan women. Am Rev Respir Dis 1992; 146:1439-44.
4. Guelar A, Gatell JM, Verdejo J, et al. A prospective study of the risk of tuberculosis among HIV- infected patients. AIDS 1993; 7:1345-9.
5. Antonucci G, Girardi E, Raviglione MC, Ippolito G. Risk factors for tuberculosis in HIV-infected persons, a prospective cohort study. JAMA 1995; 274: 143-8
6. Hawken MP, Meme HK, Elliott LC, et al. Isoniazid preventive therapy for tuberculosis in HIV-1 infected adults: results of a randomized controlled trial. AIDS 1997; 11:875-82.
7. Whalen CC, Johnson JL, Okwera A, Hom DL, et al. A trial of three regimens to prevent tuberculosis in Ugandan adults infected with the Human Immunodeficiency Virus. N Engl J Med 1997; 337:801-8.
8. Mwinga AG, Hosp M, Godfrey-Faussett P, et al. Twice weekly tuberculosis preventative therapy in HIV infection in Zambia. AIDS 1998; 12: 2447-2457.
9. Wilkinson D, Squire SB, Garner P. Effect of preventive treatment for tuberculosis in adults infected with HIV: systematic review of randomised placebo controlled trials. BMJ 1998; 17:625-629.
10. Snider DE. Pyridoxine supplementation during isoniazid therapy. Tubercle 1980;61:191-6.
11. WHO/ UNIAIDS. Policy Statement on preventive therapy against tuberculosis in people living with HIV. 1998; WHO/TB/98.255

12. Quigley M, A Mwinga, Hosp M, Lisse I, Fuchs D, Porter JDH, Godfrey-Faussett P.. Long term effect of preventive therapy for tuberculosis in a cohort of HIV-infected Zambian adults. AIDS 2001; 15: 215-222.

13. HIV sentinel surveillance in Botswana. Ministry of Health. NACP, Botswana, July 2000.

14. Botswana National TB Programme Report, 1999. Ministry of Health, Gaborone, Botswana.

15. Lockman S, N Hone, TA Kenyon, et al. Etiology of pulmonary infections in adults hospitalized with suspected tuberculosis, Botswana. [submitted to Int J Tuberc Lung Dis May 2001]

16. Oyewo T.A., E.A. Talbot, T.L. Moeti, N.J. Binkin, J.W. Tappero, T.A. Kenyon. Non-response to antibiotics predicts tuberculosis in AFB-smear-negative TB suspects, Botswana, 1997-1999. International Union Against Tuberculosis and Lung Disease Conference, Nov 1-5, 2001, Paris, France [submitted].

17. N. A. Ansari, A.H. Kombe, T.A. Kenyon, N.M. Hone, J.W. Tappero, S. Nyirenda, N.J. Binkin, S.B. Lucas. Pathology and Causes of Death in a Group of 128 Predominantly HIV-Positive Patients in Botswana, 1997-1998. [Submitted to AIDS Dec 2000]

18. Kenyon TA, Copeland JE, Moeti TL, Oyewo TA, Binkin NJ. Transmission of *Mycobacterium tuberculosis* among employees in a US Government office, Gaborone, Botswana. Int J Tuberc Lung Dis 2000; 4(10):962-7.

19. Lockman S, Tappero J, Kenyon T, et al. Tuberculin reactivity in a population of children with high BCG vaccination coverage, Botswana. Int J Tuberc Lung Dis 1999;3:23-30.

20. Duncan LE, Elliott AM, Hayes RJ et al. Tuberculin sensitivity and HIV-1 status of patients attending a sexually transmitted disease clinic in Lusaka, Zambia: a cross-sectional study. Trans R Soc Trop Med Hyg 1995; 89:37-40.

21. CDC. Tuberculin reactions in apparently health HIV-seropositive and HIV-seronegative women - Uganda. MMWR 1990; 39:638-639, 645-646.

22. Talbot E.A., T.A. Kenyon, M. Mwasekaga, T. Moeti, V. Mallon, N.J. Binkin. Control of drug resistant TB, Botswana, 1999. Africa IUATLD, 26-28 May 2000, Conakry, Guinea.

23. Mosimaneotsile B., Moalosi G., T.L. Moeti, E. Lloyd, E.A. Talbot, E.J. Lee, T.A. Kenyon. Validation of the screening algorithm for isoniazid preventive therapy (IPT) for HIV-infected persons, Botswana, 2000-2001. International Union Against Tuberculosis and Lung Disease Conference, Nov 1-5, 2001, Paris, France [submitted].

24. Karnofsky DA: The Clinical Evaluation of Chemotherapeutic Agents in Cancer. New York, Columbia University Press, 1949

25. CDC. 1993 Revised classification system for HIV infection and expanded surveillance case definition for AIDS among adolescents and adults. MMWR 1992;41:RR17.

26. Knoben, J., and Anderson, P., eds. (1988). Handbook of Clinical Drug Data (6th ed.). Illinois: Drug Intelligence Publications.

27. Gilman, A., Rall, T., Nies, A., and Taylor, P., eds. (1990). The Pharmacological Basis of Therapeutics (8th ed.). New York: Pergamon.

28. CDC. Prevention and treatment of tuberculosis among patients infected with human immunodeficiency virus: princliples of therapy and revised recommendations. MMWR 1998; 47(no. RR-20): 37.

29. Horwitz O, Bunch-Christensen K. Correlation between tuberculin sensitivity after two months and five years among BCG vaccinated subjects. Bull World Health Organ 1972; 47: 49-58.

30. van Embden J, Cave MD, Crawford JT, et al. Strain identification of

Mycobacterium tuberculosis by DNA fingerprinting: recommendations for a standardized methodology. J Clin Microbiol 1993; 31: 406-409.

31. UNDP. Botswana Human Development Report 1997. TA Publications (Botswana) Pty Ltd 1998: 33. 32. Prochaska JO, Norcross JC, DiClemente CC. Changing for Good. New York: William Morrow and Company, Inc.;1994.

33. Mosimaneotsile B, Talbot EA, Moeti TL, et al. Value of chest radiography in a tuberculosis prevention programme for HIV-infected people, Botswana. Lancet 2003; 362:1551-1552.

34. Thio C, Seaberg EC, Skolasky R, et al. HIV-1, hepatitis B virus and risk of liver-related mortality in the Multicenter Cohort Study (MACS). Lancet 2002; 360:1921-1926.
